# Relative susceptibility to pesticides and environmental conditions of *Frankliniella intonsa* and *F*. *occidentalis* (Thysanoptera: Thripidae), an underlying reason for their asymmetrical occurrence

**DOI:** 10.1371/journal.pone.0237876

**Published:** 2020-08-20

**Authors:** Mohammad Mosharof Hossain Bhuyain, Un Taek Lim

**Affiliations:** 1 Department of Entomology, Faculty of Agriculture, Hajee Mohammad Danesh Science and Technology University (HSTU), Dinajpur, Bangladesh; 2 Department of Plant Medicals, Andong National University, Andong, Republic of Korea; Chinese Academy of Agricultural Sciences Institute of Plant Protection, CHINA

## Abstract

To explain the asymmetrical abundance of native *Frankliniella intonsa* (Trybom) (Thysanoptera: Thripidae) and invasive *Frankliniella occidentalis* (Pergande) in the fields, we examined differential susceptibility to pesticides and environmental conditions, i.e., nine combinations of temperatures and relative humidities (RHs). We found adult female *F*. *intonsa* to be more susceptible to most of the tested insecticides as compared to *F*. *occidentalis*. Chlorfenapyr was most toxic to both thrips’ species. In the evaluation of environment conditions in the adult stage, *F*. *intonsa* survived 2.5 and 2.4-fold longer as RH increased at 20 and 25 °C, respectively, whereas *F*. *occidentalis* survived 1.8 and 1.6-fold longer, respectively. In both pupal and larval stage, no significant effect of interaction of temperatures and RHs was found between the two species. In conclusion, the insecticides tested differed considerably in their species-specific toxicity, and *F*. *intonsa* was generally more susceptible to the insecticides, while at the same time survivorship was better at higher RH conditions than *F*. *occidentalis*. Thus, differences in the relative susceptibility to changing environmental conditions, especially humidity, may be an underlying mechanism for the recent dominance of *F*. *intonsa* over *F*. *occidentalis* in the strawberry plastic greenhouse in Korea.

## Introduction

Both *Frankliniella intonsa* (Trybom) (Thysanoptera: Thripidae) and *Frankliniella occidentalis* (Pergande) are economically important pests of vegetables, fruits, and ornamental crops [1]. They cause damage by direct feeding on plant sap as well as transmitting tospoviruses, such as TSWV, INSV, and GRSV [2]. In recent field observations, we found that *F*. *intonsa* was the regionally dominant species in Korea over *F*. *occidentalis* had been considered as a major species in Korea [3]. In addition to pre-existing explanation, i.e., interspecific competition, for the asymmetrical occurrence, we hypothesized that differential pesticide susceptibility or environmental tolerances may be an underlying reason for the phenomenon.

Currently, application of insecticides is the most common tool for the control of those thrips, although pesticide treatment is not always effective due to thrips’ small size and cryptic behavior, high reproductive rates, and rapid development of insecticide resistance [1, 4, 5]. Indeed, insecticide resistance by *F*. *occidentalis* has been reported in a wide range of chemical classes such as organochlorines, organophosphates, carbamates, pyrethroids, and spinosyns [5–9]. However, insecticide susceptibility of *F*. *intonsa* has rarely been reported. Potential difference in susceptibility to insecticide between the two species could affect the interspecific relationship of these two sympatric species [10].

Among environmental factors, temperature or relative humidity (RH) are considered to be the most important as they affect many biological parameters such as development and reproduction [11–14] as well as population dynamics [15, 16] of insects. A close relationship exists between the physiological needs of insects and the climatic conditions of the environment. For the better decision of pest management tactics against thrips, it is essential to know their seasonal occurrence under varying temperature and humidity conditions. Seasonal occurrence has been predicted by different researchers using different models to determine how temperature affects thrips development and reproduction [17, 18]. But, there have been relatively less number of studies addressed the effect of humidity on thrips. Kakei and Tsuchida [19] reported that increasing humidity reduced pupal mortality of *Thrips palmi* (Karny). Fatnassi et al. [20] found the dependence of thrips infestation on micro-climate factors including humidity in a rose greenhouse. More interestingly, Garrick et al. [21] suggested that higher fecundity of native *Frankliniella bispinosa* (Morgan) at high humidity level can enhance biotic resistance against invasive *F*. *occidentalis*. However, no comparative data have been published on the interactive effect of temperature and RH on *F*. *intonsa* and *F*. *occidentalis*.

Therefore, we designed our experiments to investigate the relative susceptibility of these two thrips to different pesticides as well as their responses to difference combinations of temperature and RH, to help explain distribution and occurrence patterns of these thrips in the field.

## Materials and methods

### Thrips rearing

Both species of thrips were reared as described by Mainali and Lim [22], at 24 °C and 16:8 (L:D) h photoperiod in a growth chamber (DS-11 BL, Dasol Scientific Co. Ltd., Hawseong, Republic of Korea). Briefly, thrips were collected in 2012 from a strawberry greenhouse in Songcheon, Andong, Korea, and were reared in ventilated plastic containers (24 × 17 × 8 cm^3^) on leaves of eight rooted red kidney bean plants (*Phaseolus vulgaris* [L.]) wrapped in water-soaked cotton. A mixture of honey and pine pollen was streaked along the mid rib of each leaf, and water was added to the cotton whenever needed during the rearing period. Thirty adult females of either *F*. *occidentalis* or *F*. *intonsa* were used for the production of the next generation and to maintain the colony.

### Lethal effects of pesticides on adult *F*. *intonsa* and *F*. *occidentalis*

#### By direct spray

Four insecticides, one fungicide, and one herbicide (Table 1) were selected for the tests. Five 1–3 day-old adult females of either *F*. *intonsa* or *F*. *occidentalis* were placed in a centrifugal tube (50 mL capacity) with the bottom was removed and re-covered with mesh cloth to prevent the thrips drowning after pesticide spray. Each pesticide was diluted with distilled water to make 200 mL solution of the recommended field-rate and each pesticide was then sprayed (2.0 mL) onto a group of thrips using a hand sprayer (360 mL cap.). A control group was sprayed with distilled water and assessed in the same manner as above. After pesticide application, each replicate group (five thrips) was transferred using a paint brush into a Petri dish (5.0 D × 1.3 H cm) whose lid had single hole for ventilation covered with mesh and kept at 24 °C, 70–75% RH and a 16 L: 8 D h photoperiod in a growth chamber (DS-11 BL, Dasol Scientific Co. Ltd.) without food sources. The RH was set according to Winston and Bates [23] using saturated salt solutions of NaCl. The thrips mortality was recorded hourly for the insecticides tested, but for the fungicide, herbicide, or control it was recorded hourly up to 5 hours, then at 2, 4, and 8 hours’ intervals. Replications were conducted with 10 times (50 females) for each pesticide. The temperatures and RHs were recorded hourly using the Hobo data logger (H8-003-02, Onset computer corporation, Bourne, MA, USA).

**Table 1:** List of pesticides used for the bioassay of lethal effect against *Frankliniella intonsa* and *Frankliniella occidentalis* in laboratory.

Use type	Class	Active ingredient (Common name)	Trade name (Formulation type)	Company	Field recommended rate (per 20 liter of H_2_O)
Insecticide	Chlorfenapyr	Chlorfenapyr	Secure (SC)	Dongbu Hannong	20 mL
	Organophosphate	Chlorpyrifos	Dursban (WP)	Dongbu Hannong	20 g
	Neonicotinoid	Thiamethoxam	Actara (WG)	Syngenta Korea	10 g
	Microbial	Spinosad	Orgami (WG)	Dongbang Agro	10 mL
Herbicide	Chloroacetanilide	S-metolachlor	Superjanggun (EC)	Dongbu Hannong	60 cc
Fungicide	Xylylalanine	Metalaxyl	Ridomil (WP)	Sungbo	40 g

#### By residue on substrate

To test the effect of contact by thrips with dried pesticide residues on surfaces, the bottoms of Petri dishes (5.0 D × 1.3 H cm) were sprayed with each pesticide (2.0 mL/Petri dish), as described above in the direct spray assay, with field-recommended concentrations of each material, and allowed to dry for one hour. Five 3–7 days old female adults of each species were then placed in each Petri dish with pesticide residue and the set up was incubated in the same manner as described in the previous experiment in a growth chamber. The mortality of both thrips’ species was recorded as described above. There were 10 replicates of each treatment, for a total of 50 thrips per pesticide.

#### By oral ingestion

Females (3–7 days old) of *F*. *intonsa* or *F*. *occidentalis* were starved for 4 hours and then placed individually in Petri dishes with an insecticide-contaminated diet described by Alim and Lim [24]. The diet was prepared by mixing 5.0 mL of honey, 9.95 mL of distilled water, and 0.05 mL of each pesticide solution. The Petri dishes were kept in a growth chamber as mentioned above, and the mortality of both species was recorded over time, as in the direct spray application. A total of 50 adult thrips species were tested for each pesticide. Thrips in the control group were fed honey mixed with distilled water.

### Effects of temperatures and RHs on *F*. *intonsa* and *F*. *occidentalis*

#### Adult assay

Unmated, fully fed (on honey and pollen mixture, and bean leaf) adult females from both species (<3 days old) were taken from the laboratory colony for the experiment. Individuals (of either species) were placed separately in Eppendorf tubes (2.0 mL capacity) whose lids were covered with mesh cloth. Tubes containing thrips (without food or water) were placed inside humidity chambers (4202–0000, Bel-Art Products, Pequannock, NJ) and held at one of three different RH levels. The RHs were prepared according to Winston and Bates [23] using saturated salt solutions of Mg (NO_3_)_2_.6H_2_O, NaCl, and K_2_SO_4_ for 50–55, 70–75, and 90–95% RH, respectively. The prepared RHs chamber was then placed inside incubators set at 20, 25, or 30 °C and a 16:8 (L:D) h photoperiod, in a fully factorial design.

Fifty insects (n = 50) were tested under each treatment, and mortality was recorded every 8 h until all insects had died, and data were used to calculate median lethal time (LT_50_, h).

#### Pupae assay

Prepupae (<1 day old) of *F*. *intonsa* or *F*. *occidentalis* were taken from the laboratory rearing colony and placed, using a paint brush, into a Petri dish (5 cm dia.). Other procedures were followed as mentioned above. In each combination (n = 50), data were recorded every 12 hours to calculate pupal mortality.

#### Larval assay

First instar larvae (<8 h old) of *F*. *intonsa* or *F*. *occidentalis* were taken from the laboratory rearing colony and placed, using a water-soaked paint brush, into a Petri dish (5 cm dia.). Other procedures were followed as mentioned above. In each combination (n = 50), data were recorded every 2 hours until either death or pupation to calculate LT_50_ (h).

### Statistical analysis

Mortality data on the lethal effects for pesticides and the effects of environmental conditions were analyzed with the Chi-square tests, and LT_50_ values were calculated using probit analysis [25]. Significant differences among pesticides or environmental treatments were recorded when 95% confidence intervals (CI) did not overlap.

## Results

### Lethal effects of pesticides on *F*. *intonsa* and *F*. *occidentalis*

**Fig 1:**
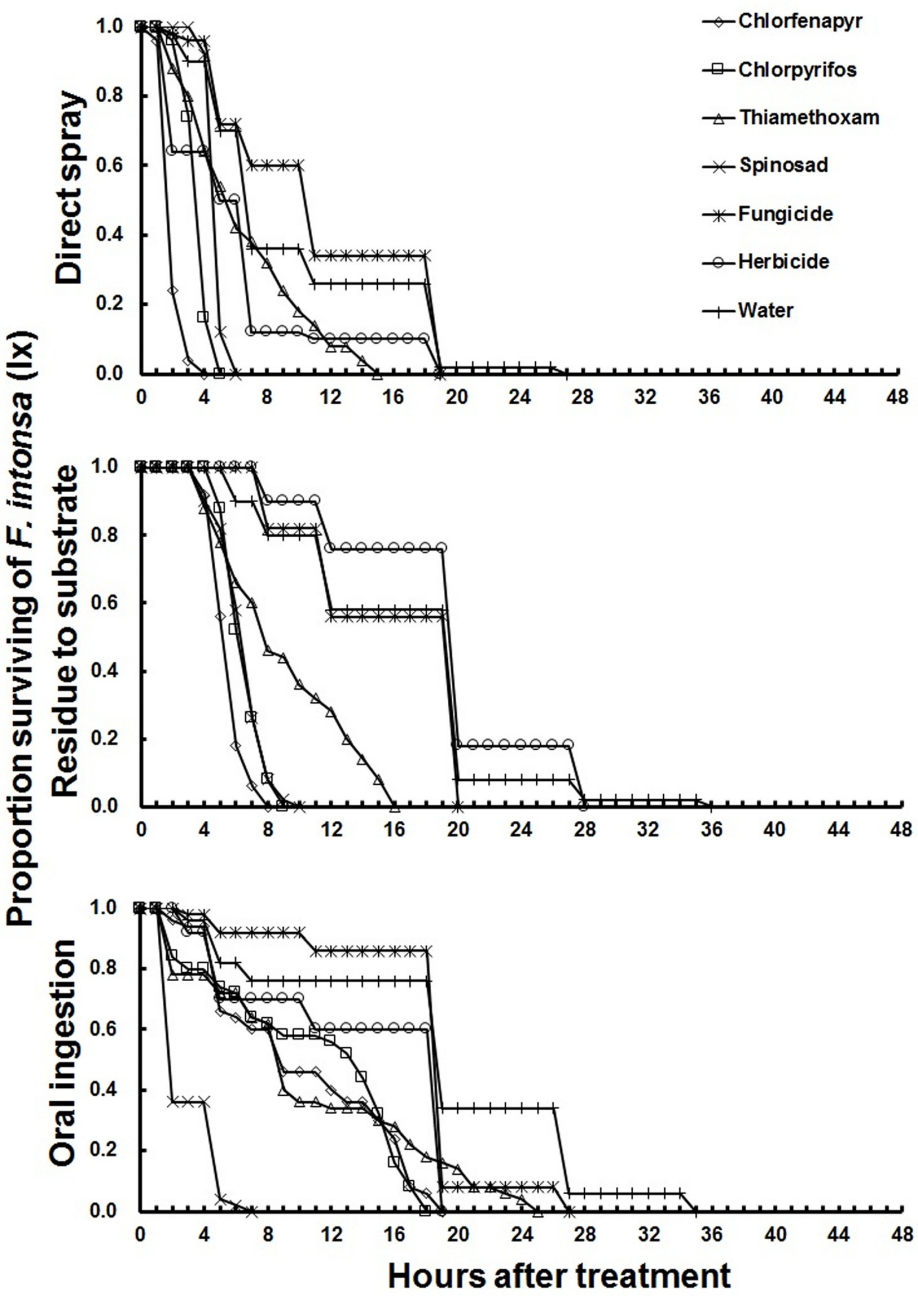
Relative susceptibility of adult females *Frankliniella intonsa* to different pesticides after direct spray, residue to substrate and oral ingestion under laboratory condition.

Results of acute toxicity assays with six pesticides showed *F*. *intonsa* to be highly susceptible to chlorfenapyr, both as a direct spray and as a residue on substrate, with all test individuals dying within 9 hours of treatment (Fig 1, Table 2). The LT_50_ of chlorfenapyr, chlorpyrifos, thiamethoxam, and spinosad were significantly lower than those of s-metolachlor or metalaxyl, regardless of exposure methods, except for exposure to metalaxyl via direct spray application (Table 2).

**Table 2:** Statistical comparison of the lethal effects of six pesticides on adult female *Frankliniella intonsa* (n = 50).

Assayed by	Pesticide	LT_50_ (h)	95% CI (lower limit, upper limit)	Slope ± SE	χ^2^ (df)
Direct spray	Chlorfenapyr	1.66a	(1.50, 1.81)	7.62 ± 0.91	0.81 (2)
	Chlorpyrifos	3.26b	(2.74, 3.76)	10.82 ± 1.30	6.81 (3)
	Thiamethoxam	5.23d	(4.80, 5.64)	3.51 ± 0.24	8. 07 (13)
	Spinosad	4.52c	(4.38, 4.65)	26.76 ± 3.48	0.03 (4)
	S-metolachlor	9.95f	(8.91, 11.10)	2.98 ± 0.21	34.57 (17)
	Metalaxyl	4.13bcd	(3.33, 4.87)	2.59 ± 0.17	41.63 (17)
	Control (water)	7.64e	(6.76, 8.47)	3.37 ± 0.17	61.05 (25)
Residue to substrate	Chlorfenapyr	4.12a	(3.91, 4.32)	10.45 ± 1.07	0.80 (5)
	Chlorpyrifos	5.14b	(4.91, 5.35)	11.22 ± 1.11	1.78 (6)
	Thiamethoxam	6.82c	(6.36, 7.28)	3.85 ± 0.27	11.83 (13)
	Spinosad	4.93b	(4.56, 5.29)	8.29 ± 0.71	9.98 (7)
	S-metolachlor	14.81de	(13.21, 17.35)	4.11 ± 0. 34	59.78 (17)
	Metalaxyl	16.79e	(15.48, 18.24)	5.42 ± 0.30	106. 46 (25)
	Control (water)	13.27d	(12.17, 14.33)	4.78 ± 0.20	118.94 (33)
Oral ingestion	Chlorfenapyr	8.70b	(7.81, 9.61)	3.30 ± 0.22	34.96 (17)
	Chlorpyrifos	9.44b	(7.75, 11.67)	2.31 ± 0.18	63.36 (16)
	Thiamethoxam	7.77b	(6.81, 8.70)	2.53 ± 0.14	46.51 (23)
	Spinosad	2.31a	(1.14, 3.21)	4.20 ± 0.40	34.20 (5)
	S-metolachlor	16.44c	(12.17, 22.97)	5.38 ± 0.27	954.73 (25)
	Metalaxyl	15.61c	(12.38, 23.12)	1.97 ± 0.19	65.10 (17)
	Control	15.94c	(14.12, 17.87)	3.37 ± 0.16	177.46 (33)

Means followed by different letter(s) are significantly different at each pesticide.

**Fig 2:**
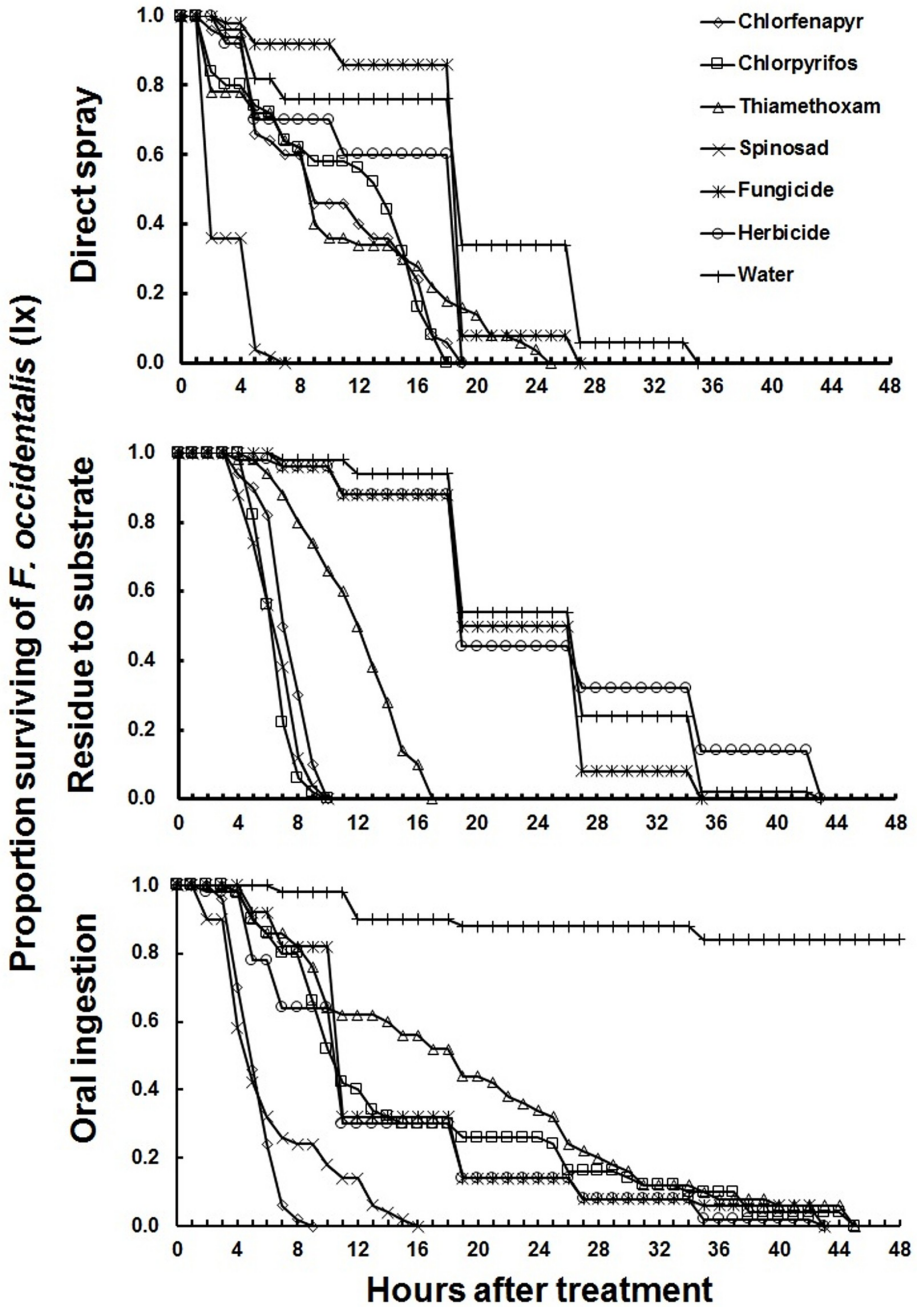
Relative susceptibility of adult females *Frankliniella occidentalis* to different pesticides after direct spray, residue to substrate and oral ingestion under laboratory condition.

As with *F*. *intonsa*, chlorfenapyr was highly toxic to *F*. *occidentalis*, and 100% mortality occurred within 12 hours of treatment by either direct spray, exposure to residue, and oral ingestion (Fig 2 and Table 3). The LT_50_ of chlorfenapyr, chlorpyrifos, thiamethoxam, and spinosad were significantly lower than those of s-metolachlor or metalaxyl, regardless of exposure methods (Table 3).

**Table 3:** Statistical comparison of the lethal effects of six pesticides on adult female *Frankliniella occidentalis* (n = 50).

Assayed by	Pesticide	LT_50_ [h]	95% CI (lower limit, upper limit)	Slope ± SE	χ^2^ (df)
Direct spray	Chlorfenapyr	8.76b	(7.87, 9.67)	3.25 ± 0.21	33.98 (17)
	Chlorpyrifos	9.44b	(7.75, 11.67)	2.31 ± 0.18	63.36 (16)
	Thiamethoxam	7.77b	(6.81, 8.70)	2.53 ± 0.14	46.51 (23)
	Spinosad	2.31a	(1.14, 3.21)	4.20 ± 0.40	34.20 (5)
	S-metolachlor	16.44c	(12.17, 22.97)	5.38 ± 0.27	954.73 (25)
	Metalaxyl	15.61c	(12.38, 23.12)	1.97 ± 0.19	65.10 (17)
	Control (water)	15.94c	(14.12, 17.87)	3. 37 ± 0.16	177.46 (33)
Residue to substrate	Chlorfenapyr	6.85b	(6.38, 7.34)	9.65 ± 0.84	16.60 (8)
	Chlorpyrifos	6.08a	(5.86, 6.30)	12.72 ± 1.17	1.63 (8)
	Thiamethoxam	11.03d	(10.45, 11.64)	6.52 ± 0.45	22.28 (15)
	Spinosad	5.99a	(5.72, 6.26)	8.60 ± 0.73	8.00 (8)
	S-metolachlor	20.87e	(19.52, 22.28)	6.14 ± 0.28	146.52 (33)
	Metalaxyl	22.66e	(21.56, 23.78)	4.43 ± 0.19	76.51 (41)
	Control (water)	23.00e	(21.70, 24.30)	6.70 ± 0.27	175.88 (41)
Oral ingestion	Chlorfenapyr	4.76a	(4.52, 5.00)	6.66 ± 0.77	2.11 (7)
	Chlorpyrifos	12.20c	(11.49, 12.89)	3.08 ± 0.13	35.36 (43)
	Thiamethoxam	15.62d	(14.87, 16.36)	3.32 ± 0.13	30.30 (43)
	Spinosad	4.99a	(4.52, 5.44)	3.62 ± 0.24	14.97 (14)
	S-metolachlor	12.14c	(11.32, 12.94)	3.64 ± 0.15	63.02 (41)
	Metalaxyl	10.25b	(9.62, 10.86)	3.23 ± 0.13	33.44 (41)
	Control	175.00e	(170.49, 596.90)	1.16 ± 0.15	17.92 (46)

Means followed by different letter(s) are significantly different at each pesticide.

Chlorfenapyr’s LT_50_ was lower (i.e., the compound was more toxic) for *F*. *intonsa* than *F*. *occidentalis* by both direct spray and residual assays, but in the oral ingestion assay, the LT_50_ of chlorfenapyr was lower in *F*. *occidentalis* (Tables 2 and 3). Thiamethoxam had a lower LT_50_ in *F*. *intonsa* than *F*. *occidentalis* in all assays. In the test of spinosad, the LT_50_ was lower in *F*. *intonsa* than *F*. *occidentalis* in both residual and oral ingestion assay, but lower in *F*. *occidentalis* than *F*. *intonsa* in direct spray application. Both the fungicide and herbicide showed lower LT_50_ in *F*. *intonsa* than *F*. *occidentalis* in both direct spray and residual assay, but response was opposite when applied via oral ingestion (Tables 2 and 3).

### Effects of temperatures and RHs on *F*. *intonsa* and *F*. *occidentalis*

**Fig 3:**
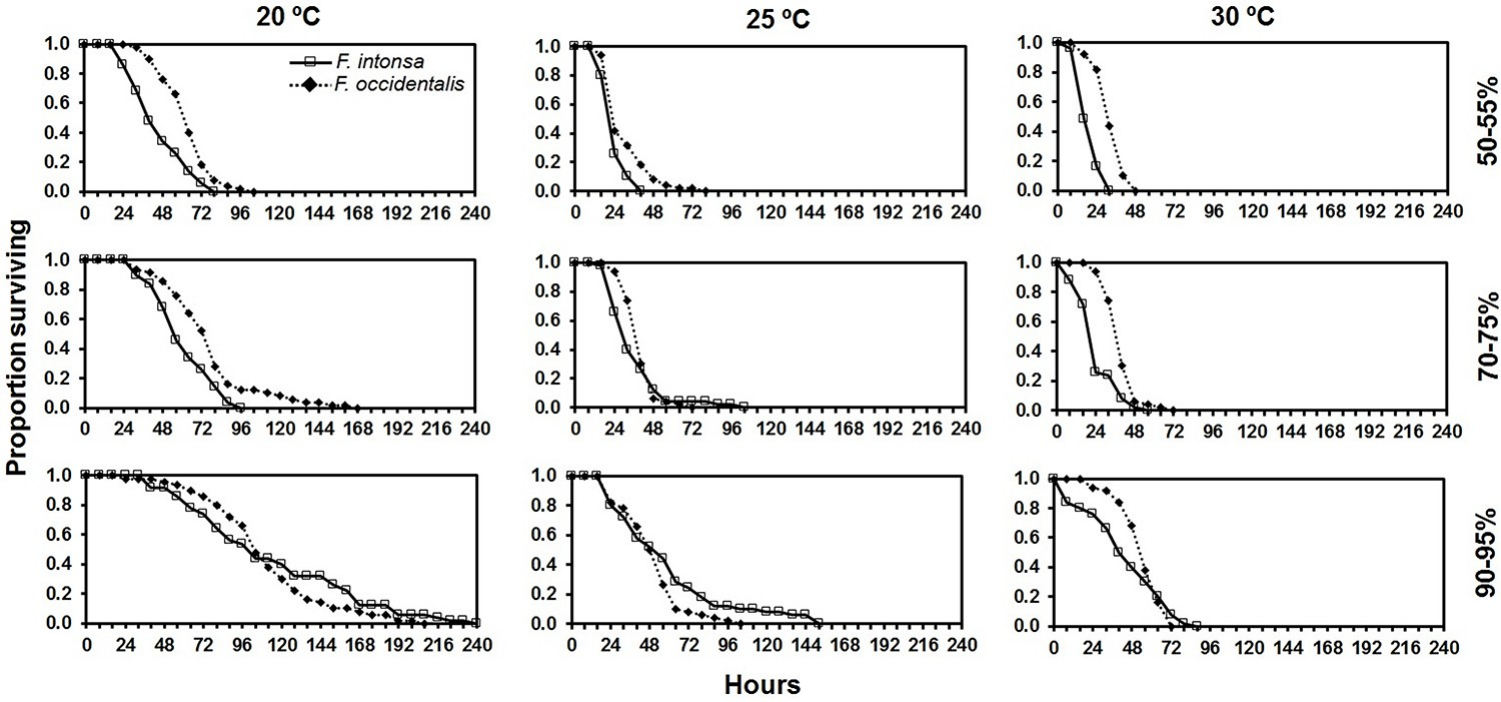
Proportion surviving of *Frankliniella intonsa* and *Frankliniella occidentalis* adults (female) to various exposures times at different interactive temperature and relative humidity conditions.

Adult survivorship of both *F*. *intonsa* and *F*. *occidentalis* decreased with increasing temperature, and the longest survival was found at 20 °C in both thrips’ species (Fig 3). The LT_50_ of adult *F*. *occidentalis* were longer (indicating higher survivorship) than that of *F*. *intonsa* in all the tested combinations of temperatures and RHs except both 20 and 25 °C at high humidity (Fig 3, Table 4). The adult survivorship of both thrips species generally increased with increasing RH (Fig 3). There was an increase in LT_50_ of 2.5 and 2.4-fold for *F*. *intonsa* and 1.8 and 1.6-fold for *F*. *occidentalis* when RH increased from low to high at 20 and 25 °C, respectively.

**Table 4:** Median lethal time [LT_50_ (h)] of adult females *Frankliniella intonsa* (n = 50) and *Frankliniella occidentalis* (n = 50) at different environmental conditions.

Temperature (°C)	Relative humidity (%)	Species	LT_50_ (h)	95% CI (lower limit, upper limit)	Slope ± SE	χ^2^ (df)
20	50–55	*F*. *intonsa*	39.61a	(37.12, 42.04)	5.70 ± 0.46	5.20 (8)
		*F*. *occidentalis*	58.08b	(55.77, 60.34)	9.15 ± 0.67	5.16 (11)
	70–75	*F*. *intonsa*	54.55b	(51.97, 57.10)	7.06 ± 0.54	7.53 (10)
		*F*. *occidentalis*	68.53d	(65.40, 71.58)	6.13 ± 0.34	12.99 (19)
	90–95	*F*. *intonsa*	98.86e	(94.47, 103.18)	4.59 ± 0.22	16.34 (28)
		*F*. *occidentalis*	100.03e	(83.29, 115.94)	6.21 ± 0.30	365.55 (24)
25	50–55	*F*. *intonsa*	20.43a	(18.86, 21.89)	7.79 ± 0.91	1.94 (3)
		*F*. *occidentalis*	25.91b	(23.29, 28.33)	5.36 ± 0.45	8.25 (8)
	70–75	*F*. *intonsa*	30.11b	(27.73, 32.36)	5.13 ± 0.38	10.67 (11)
		*F*. *occidentalis*	38.50c	(35.53, 41.40)	7.15 ± 0.61	8.94 (7)
	90–95	*F*. *intonsa*	47.08de	(43.54, 50.49)	3.67 ± 0.22	9.04 (17)
		*F*. *occidentalis*	42.74ce	(39.39, 45.96)	5.66 ± 0.39	14.74 (11)
30	50–55	*F*. *intonsa*	15.67a	(11.81, 19.16)	6.32 ± 0.74	2.08 (2)
		*F*. *occidentalis*	28.73b	(23.66, 33.88)	7.80 ± 0.79	13.00 (4)
	70–75	*F*. *intonsa*	18.51a	(14.57, 22.18)	4.14 ± 0.38	10.11 (5)
		*F*. *occidentalis*	35.79c	(34.00, 37.51)	9.74 ± 0.89	3.79 (7)
	90–95	*F*. *intonsa*	32.46bc	(23.46, 41.64)	2.91 ± 0.23	45.66 (9)
		*F*. *occidentalis*	49.47d	(43.14, 56.92)	7.78 ± 0.71	31.40 (7)

Means followed by different letter(s) are significantly different at each temperature

As with adults, humidity positively affected pupal survivorship of the both thrips species, and pupal mortality decreased with increasing RH at all temperatures tested (20 °C *χ*^*2*^ = 52.74, *df* = 5, *P* < 0.001; 25 °C *χ*^*2*^ = 20.24, *df* = 5, *P* < 0.001; 30 °C *χ*^*2*^ = 27.04, *df* = 5, *P* < 0.001) (Table 5). Different from adults, there was no interspecific difference in pupal mortality in any combinations of temperatures and RHs.

**Table 5:** Pupal mortality of *Frankliniella intonsa* and *Frankliniella occidentalis* at different environmental conditions (n = 50).

Temperature (°C)	Relative humidity (%)	Species	Mortality
20	50–55	*F*. *intonsa*	0.40a
		*F*. *occidentalis*	0.42a
	70–75	*F*. *intonsa*	0.12b
		*F*. *occidentalis*	0.06b
	90–95	*F*. *intonsa*	0.06b
		*F*. *occidentalis*	0.04b
25	50–55	*F*. *intonsa*	0.34ab
		*F*. *occidentalis*	0.36a
	70–75	*F*. *intonsa*	0.26ab
		*F*. *occidentalis*	0.28ab
	90–95	*F*. *intonsa*	0.06c
		*F*. *occidentalis*	0.12bc
30	50–55	*F*. *intonsa*	0.34ab
		*F*. *occidentalis*	0.44a
	70–75	*F*. *intonsa*	0.16bc
		*F*. *occidentalis*	0.34ab
	90–95	*F*. *intonsa*	0.14bc
		*F*. *occidentalis*	0.08c

Mean values with the same letter (s) in each temperature are not statistically different.

**Fig 4:**
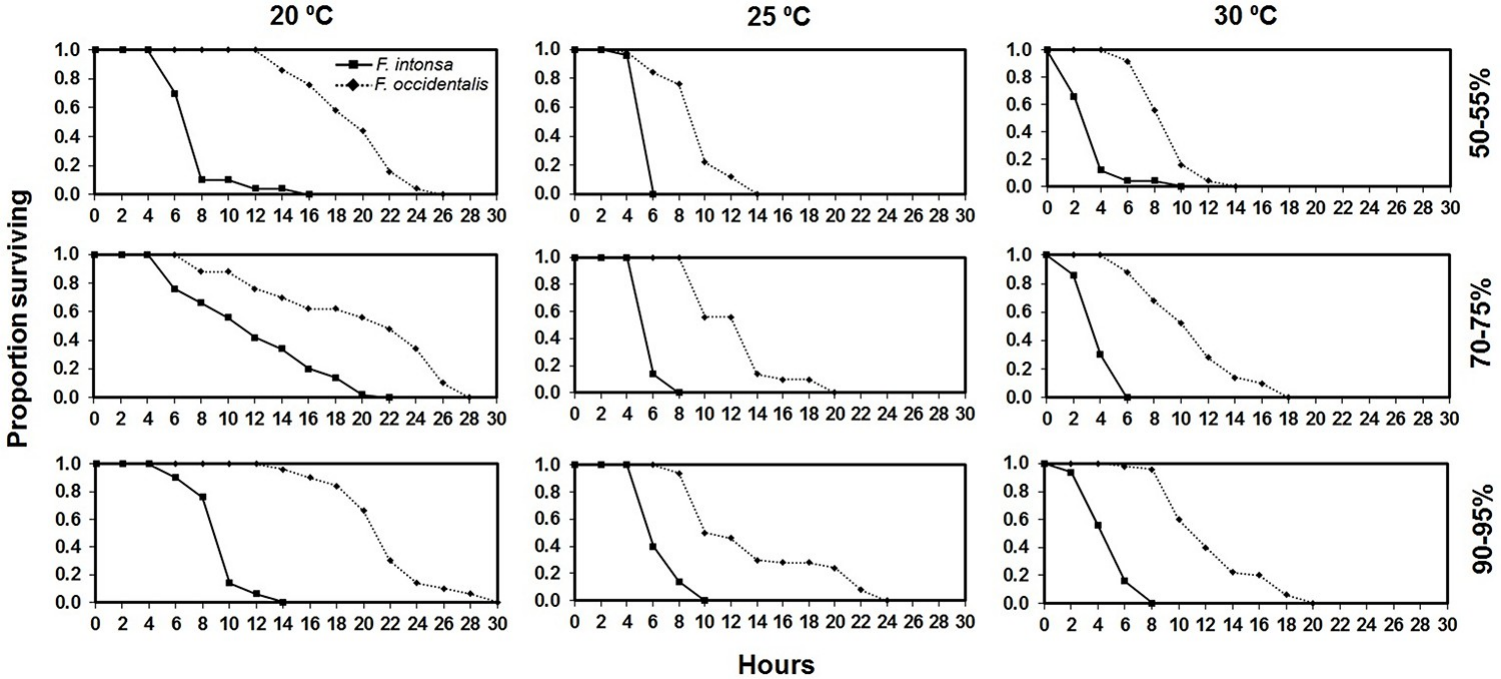
Proportion surviving of *Frankliniella intonsa* and *Frankliniella occidentalis* larvae (1^st^ instar) to various exposures times at different interactive temperature and relative humidity conditions.

Considering all temperatures and RHs, larval *F*. *occidentalis* survived better than *F*. *intonsa* (Fig 4). The LT_50_ of larval *F*. *occidentalis* larvae were longer than *F*. *intonsa* at all the tested temperatures and RHs except three pairs that were not analyzed due to data heterogeneity (Table 6).

**Table 6:** Median lethal time [LT_50_ (h)] of larval (1^st^ instar) *Frankliniella intonsa* (n = 50) and *Frankliniella occidentalis* (n = 50) at different environmental conditions.

Temperature (°C)	Relative humidity (%)	Species	LT_50_ (h)	95% CI (lower limit, upper limit)	Slope ± SE	χ^2^ (df)
20	50–55	*F*. *intonsa*	6.75a	(5.64, 7.69)	8.23 ± 1.01	11.55 (6)
		*F*. *occidentalis*	18.38d	(17.68, 19.10)	12.68 ± 1.34	6.04 (11)
	70–75	*F*. *intonsa*	10.23c	(9.32, 11.11)	4.83 ± 0.49	8.21 (9)
		*F*. *occidentalis*	18.16de	(16.18, 20.63)	4.63 ± 0.48	23.18 (12)
	90–95	*F*. *intonsa*	8.50b	(7.87, 9.11)	10.97 ± 1.43	6.42 (5)
		*F*. *occidentalis*	20.54e	(19.79, 21.27)	13.11 ± 1.26	4.26 (13)
25	50–55	*F*. *intonsa*	‒^$^	(—)	56.82 ± 0.00	0.00 (1)
		*F*. *occidentalis*	8.69b	(7.81, 9.55)	9.11 ± 1.17	5.95 (5)
	70–75	*F*. *intonsa*	‒^$^	(—)	57.48 ± 0.00	0.00 (2)
		*F*. *occidentalis*	11.64c	(10.57, 12.62)	9.31 ± 1.03	12.83 (8)
	90–95	*F*. *intonsa*	5.99a	(5.04, 6.76)	11.28 ± 1.81	3.31 (3)
		*F*. *occidentalis*	12.24c	(10.87, 13.58)	5.62 ± 0.55	16.52 (10)
30	50–55	*F*. *intonsa*	2.39a	(1.88, 2.82)	4.49 ± 0.74	2.89 (3)
		*F*. *occidentalis*	8.22b	(7.70, 8.72)	11.41 ± 1.55	1.19 (5)
	70–75	*F*. *intonsa*	‒^$^	(—)	6.47 ± 1.04	1.85 (1)
		*F*. *occidentalis*	9.62c	(8.86, 10.35)	6.42 ± 0.71	3.25 (7)
	90–95	*F*. *intonsa*	3.99a	(2.35, 5.53)	2.91 ± 0.23	2.48 (2)
		*F*. *occidentalis*	11.32d	(10.57, 12.04)	7.58 ± 0.81	4.27 (8)

^$^Calculation suspended due to heterogeneity in probit analysis. Means followed by different letter(s) are significantly different at each temperature.

## Discussion

Interspecific competition can be affected by anthropogenic factors such as global warming and farming practice [10, 15]. Species displacement by the outcome of interspecific competition, has particular relevance to pest management and conservation biology [26, 27]. Displacements among *Liriomyza* species in the past decade could be an example outcome of global warming [15]. Zhao et al. [10] demonstrated, in both laboratory and field trials, that *F*. *occidentalis* is significantly less susceptible than *T*. *tabaci* to chemical insecticides. Therefore, following application of insecticides, *F*. *occidentalis* became the predominant species, while in plots not treated with insecticides, *T*. *tabaci* remained the predominant species.

Several pesticides are recommended for use against *F*. *occidentalis*, but very few against *F*. *intonsa*. To control *F*. *occidentalis* in various crops, growers often use insecticides as the main control strategy [5, 28]. Shan et al. [29] reported chlorfenapyr, chlorpyrifos, and spinosad to be effective in controlling *F*. *occidentalis*, which was in alignment with our results. Our results indicated that chlorfenapyr was the most toxic among the six pesticides tested against *F*. *intonsa* in both the direct spray and residual assays similar to previous study [28].

We found asymmetrical sensitivity to pesticides between *F*. *intonsa* and *F*. *occidentalis*, and *F*. *intonsa* was found to be more susceptible than *F*. *occidentalis*. This indicates that *F*. *intonsa* would become a superior competitor against *F*. *occidentalis* only in the crop field that were not treated with insecticides. Similarly, Zhao et al. [10] found that less susceptible *F*. *occidentalis* became predominant species after application of insecticides by replacing more susceptible species *T*. *tabaci*, while Gao et al. [30] found *F*. *intonsa* be more susceptible than *F*. *occidentalis* to spinetoram which is similar to our present findings. However, as we tested only adult female, further verification on other life stages may be needed in the future study.

It is necessary for any species to adapt to changes in environmental conditions. The effect of temperature on biological attributes such as longevity and fecundity have been widely studied in thrips [17, 18, 31, 32], but very few studies have been reported on the interactive influence of temperature and RH on life history parameters of insects [14, 33–35]. In this study of *F*. *intonsa* and *F*. *occidentalis*, higher RH enhanced the survivorship of both species irrespective of temperature, and *F*. *occidentalis* survived longer than *F*. *intonsa* in most combinations of temperature and RH except in the high level of RH conditions at both 20 and 25 °C. In these combinations, *F*. *intonsa* showed better survivorship than *F*. *occidentalis* as RH increase, thus indicating interactive effect of temperature and RH. Although these patterns were not observed in pupal mortality and larval survivorship experiments, this may indicate that adult *F*. *intonsa* performs better at higher humidity while *F*. *occidentalis* does better at lower humidity. Nevertheless, as our study, *F*. *occidentalis* generally showed better survivorship than *F*. *intonsa* at the same temperature [36].

Significant interaction of temperature and RH was also reported in other taxa, i.e., the two species of desert fleas, *Xenopsylla conformis* (Wagner) and *X*. *ramesis* (Rothschild) (Siphonaptera: Pulicidae). The larval survivorship at both 55 and 75% RH was significantly higher at 25 °C than at 28 °C in both species, whereas temperature had no effect on the survivorship at lower humidity. Despite the absence of our tests under 55% RH, we found such a similar interaction of temperature and RH in adult *F*. *intonsa* that showed higher increase rate of 3.0 fold LT_50_ in 90–95% than 2.5 fold LT_50_ in 50–55% when temperature decreased from 30 to 25 °C. Since *F*. *occidentalis* didn’t show such interaction of temperature and RH, *F*. *intonsa* may be more sensitive to the humidity change.

Under field conditions, we observed a higher density of *F*. *intonsa* than *F*. *occidentalis* in pepper fields [3]. Although we already suggested interspecific competition as an underlying mechanism for the asymmetry, changing environment condition, especially humidity, would be another factor. *Frankliniella intonsa* can be better at relatively higher humidity just because they have lower body water retention ability. In general, it is common for smaller insects, which have a larger relative surface area per body weight, to experience higher rates of water loss [37]. Nevertheless, it might also be due to other causes such as its shorter developmental duration and higher fecundity [32].

In summary, the thrips tested here differed in their susceptibilities to the insecticides tested, and *F*. *intonsa* was generally more susceptible to the insecticides, in all exposure methods. Temperature and RH may play an important role in determining the distribution and occurrence pattern of *F*. *intonsa* and *F*. *occidentalis*. Both thrips species are able to build up under a wide range of RHs and temperatures. Adult *F*. *occidentalis* survive better in relatively lower humidity while *F*. *intonsa* performs better at high humidity. This asymmetrical survivorship in response to RH may be another explanation of the local dominance of *F*. *intonsa* over *F*. *occidentalis*. Nevertheless, the higher insecticide susceptibility of *F*. *intonsa* will likely affect its dominance against *F*. *occidentalis* in fields where chemical insecticides are often used. Their use is decreasing with the awareness of side effects of pesticides on nontarget organisms.

## Supporting information

S1 File Mortality (pesticides), mortality (temp. and humidity, adult), mortality (temp. and humidity, pupae), mortality (temp. and humidity, larvae). (XLSX)
